# Seasonal Activity of Adult Mosquitoes (Diptera: Culicidae) in a Focus of Dirofilariasis and West Nile Infection in Northern Iran

**Published:** 2018-12-25

**Authors:** Shahyad Azari-Hamidian, Behzad Norouzi, Ayoob Noorallahi, Ahmad Ali Hanafi-Bojd

**Affiliations:** 1 Research Center of Health and Environment, Guilan University of Medical Sciences, Rasht, Iran; 2 School of Health, Guilan University of Medical Sciences, Rasht, Iran; 3 Department of Disease Control and Prevention, Health Vice-Chancellorship, Guilan University of Medical Sciences, Rasht, Iran; 4 Department of Medical Entomology and Vector Control, School of Public Health, Tehran University of Medical Sciences, Tehran, Iran

**Keywords:** *Dirofilaria*, *Flavivirus*, Flaviviridae, Vectors, Iran

## Abstract

**Background:**

Mosquito-borne arboviruses such as West Nile, dengue, Rift Valley fever, and Sindbis viruses and the nematode *Dirofilaria* are reported in Iran, but there is little information on the seasonal activity of their vectors in the country. We aimed to determine the seasonal activity of adult mosquitoes (Diptera: Culicidae) in a focus of dirofilariasis and West Nile infection in Guilan Province, northern Iran.

**Methods:**

Collections were carried out using light traps in seven counties at least two times from random sites and every two weeks from a fixed site (Pareh Village, Rudbar County) during Aug–Dec 2015 and Apr–Oct 2016.

**Results:**

Overall, 16327 adult mosquitoes comprising 18 species representing seven genera were identified. The most prevalent species were *Cx. theileri* (23.59%), *Cx. tritaeniorhynchus* (20.75%), *Cx. pipiens* (19.37%), *Ae. vexans* (18.18%), *An. pseudopictus* (10.92%) and *An. maculipennis* s.l. (5.48%). *Aedes pulcritarsis* and *Cx. perexiguus* were found for the first time in Guilan Province. The active season of adult mosquitoes extended from early May to early Oct in the fixed site. There was no significant regression between the abundance of adult mosquitoes and the meteorological data during active season in the fixed site (P> 0.05, R^2^= 0.31).

**Conclusion:**

Though no significant regression between the abundance of mosquitoes and the meteorological data was observed during active season, temperature and rice fields had a great influence in starting and ending active season in the region.

## Introduction

West Nile virus (WNV) (Flaviviridae: *Flavivirus*) and its subtype Kunjin is distributed in Eurasia, Africa, North and Central America and Australia. Mosquitoes (Diptera: Culicidae), especially ornithophilic species, are the principal vectors of the virus and some virus isolations have been reported from soft and hard ticks. Wild birds, especially wetland species, are the principal vertebrate hosts, the virus has also been isolated from mammals and frogs (1, 2).

WNV infection is recorded from horses in at least 26 provinces (out of total 31) in Iran (3–5), humans (6–12) and birds (13). Guilan Province in the Caspian Sea littoral of northern Iran, with vast wetlands, is one of the foci of WNV where infections are found in humans (1.4–10%) (4, 7–8), horses (2.2–25%) (3–4) and birds (especially the common coot, the main reservoir) (62.7%) (13). Recently, the virus was found in *Aedes* (*Ochlerotatus*) *caspius* (Pallas) s.l. [*Ochlerotatus caspius* s.l.] in West Azerbaijan Province, northwestern Iran, and in *Cx. pipiens* Linnaeus in Guilan Province, northern Iran, respectively (14, 15).

Dirofilariasis is a disease caused by different species of the nematode genus *Dirofilaria* (Spirurida: Onchocercidae), especially *D. immitis* (canine or dog heartworm) and *D. repens*, transmitted by mosquitoes. The disease is cosmopolitan. The reservoirs of the nematodes are many different mammals, especially canids. Previously, human dirofilariasis (HD) was considered a rare disease, but at the present time, HD is classified as an emerging disease in some areas because the number of reported cases was dramatically increased (16).

Dirofilariasis is found in humans, dogs, wolves, jackals, foxes and cats in at least 15 provinces of Iran (17–22). Guilan Province is one of the foci of dirofilariasis, where *D. repens* infection is found in humans (17, 23) and *D. immitis* found in 4.4% (24) to 51.4% of local dogs (25, 26). *Culex theileri* Theobald is a known vector of *D. immitis* in northwestern Iran (27).

The last checklist of Iranian mosquitoes comprises 64 species and seven genera (28, 29). Subsequently, *Anopheles superpictus* Grassi includes two species in Iran based on the Internal Transcribed Spacer 2 (ITS2) sequences of rDNA (30), later listed as species A and B (31). A new species of the *Anopheles hyrcanus* group (*An. hyrcanus* spIR) was recognized from southwestern Iran, also based on ITS2 sequences (32). More recently, the occurrence of *Aedes* (*Stegomyia*) *albopictus* (Skuse) [*Stegomyia albopicta*] and *Ae.* (*Stg.*) *unilineatus* (Theobald) [*Stegomyia unilineata*] were reported in southeastern Iran and *Orthopodomyia pulcripalpis* (Rondani) in northern Iran, respectively (33–35). Overall, 30 species of mosquitoes representing seven genera were listed in Guilan Province (36).

A large amount of available data on mosquitoes in Iran is based on collections and ecology of larvae (27, 36–40 and many other references cited by aforementioned articles). Different methods of collecting adult mosquitoes, such as using light traps, aspirators, pit shelters and total catch (Pyrethrum space spray), have been used mostly in relation to anopheline vectors of malarial protozoa (41–47). There are a few published documents in the country that deal with adult sampling, especially using light traps, which include culicines (27, 48–50), but there are no studies of seasonal activity. That is why there is very little information about the seasonal activity of culicine adults in Iran.

This study was carried out by means of light traps to study the seasonal activity of mosquitoes, especially probable and proven vectors of WNV and *Dirofilaria*, in Guilan Province, northern Iran.

## Materials and Methods

### Study area

**Fig. 1.**
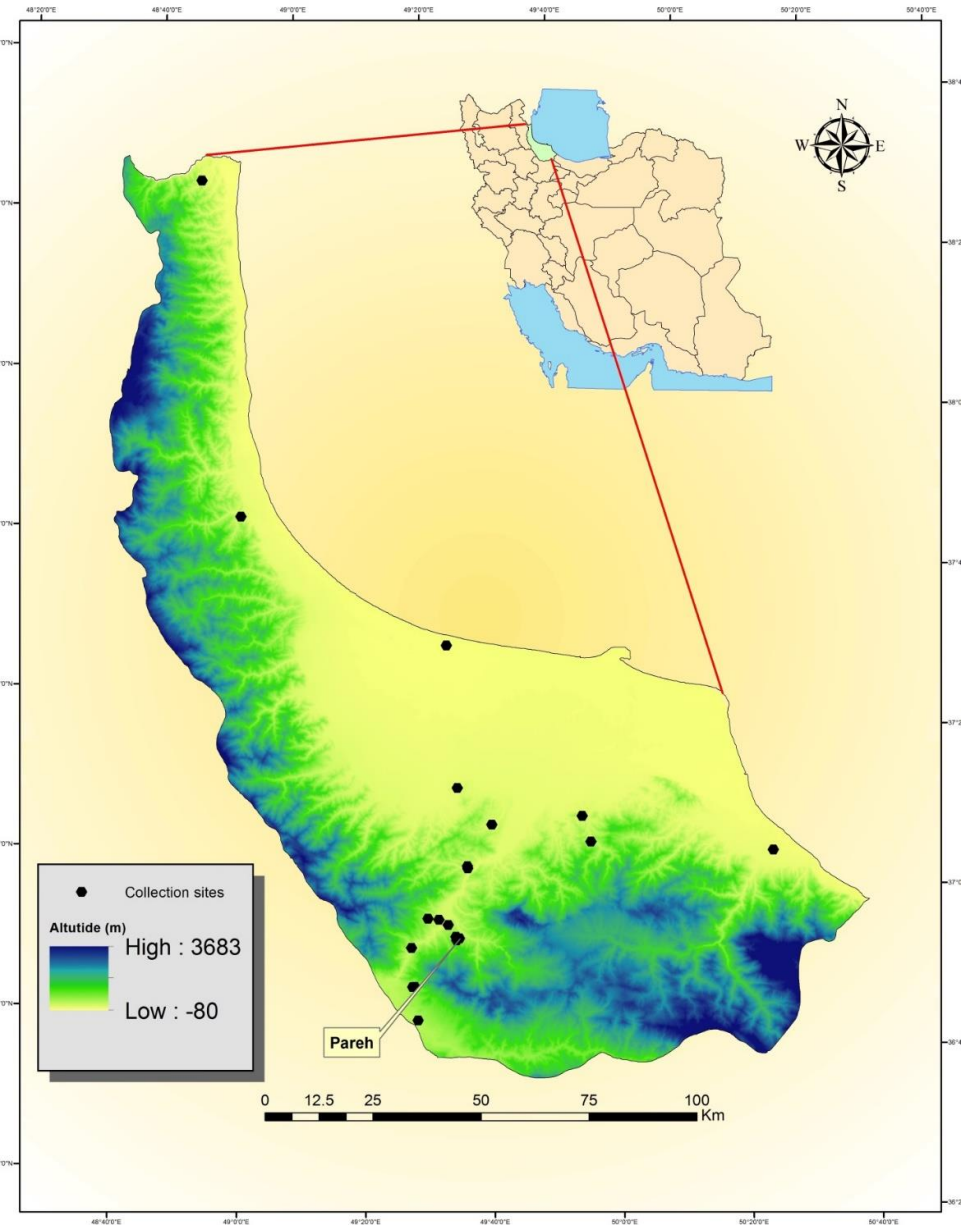
Map of Iran highlighting the location of Guilan Province including mosquito collection sites surveyed in 2015–2016

Guilan Province locates in the Caspian Sea littoral of northern Iran, between the Caspian Sea and the Alborz Mountain range. It has coastal, plain, foothill, and mountainous areas with an area of approximately 14,700 square kilometers. The province is bordered by Mazandaran Province in the east, Ardabil Province in the west and Zanjan and Qazvin provinces in the south. It is also bordered by the Republic of Azerbaijan in the north as well as Russia across the Caspian Sea (Fig. 1). The province has a temperate climate and relatively warm-humid summer. It is located between 36°33′–38°27′ N latitude and 48°32′–50°36′ E longitude and formally includes 16 counties. Most areas of Guilan Province with about 1000–2000mm of rainfall annually, have the greatest amount of rainfall in Iran, and the main agricultural crop is rice. The province has vast deciduous forests of Hyrcania, temperate climate, vast wetlands and rice fields, which provide abundant habitats for mosquitoes.

### Specimen and data collection

In seven counties (including different topographical areas of the province) adult collections were carried out at least two times from random (variable) sites during Aug–Dec 2015 and Apr–Oct 2016 (Fig. 1, Table 1). Sampling was also carried out at a fixed site (Pareh Village of Rudbar County, 36° 50.800′ N, 49° 32.650′ E, altitude 487m) every two weeks from Apr to Oct 2016. Rudbar County in southern Guilan has about 200–500mm annual rainfall and showed mountainous and less humid temperate climate similar Mediterranean Region. Pareh Village is in a foothill area and close to natural Hyrcanian forest and manmade woodland that includes trees such as olive, walnut, fig, Persian ironwood (*Parrotia persica*) and Caucasian elm or Caucasian zelkova (*Zelkova carpinifolia*). The main livelihood of the people in the village is husbandry and the main domestic animals are cattle and sheep. Dogs, horses, donkeys and fowls are also common animals in the region. The meteorological data of Pareh Village during 2016 is shown in Table 2. Two CDC light traps were used in each variable and fixed site. The light traps were suspended from the ceiling in animal shelters from sunset to sunrise, i.e. from 1800 to 0600 hrs. The electricity of traps was provided by 6-volt rechargeable batteries. Moreover, ad hoc collections were carried out using manual aspirators (hand catch) in the fixed and random sites. The specimens were identified using the morphological-based keys (29). The abbreviations of mosquito genera and subgenera follow Reinert (51). The specimens are deposited at the Museum of Medical and Veterinary Entomology, School of Health, Guilan University of Medical Sciences, Rasht, Iran.

**Table 1.** Collection data for adult mosquitoes captures at variable sites in Guilan Province, Iran, August–December 2015 and April–October 2016

**Locality (City/Village)**	**Topography**	**County**	**Coordinates**	**Altitude (m)**
**Rostamabad**	Plain	Rudbar	36° 52.999′ N, 49° 29.385′ E	215
**Joben**	Foothill	Rudbar	36° 53.072′ N, 49° 27.658′ E	399
**Khaskool**	Foothill	Rudbar	36° 50.789′ N, 49° 32.669′ E	470
**Lafandsara**	Foothill	Rudbar	36° 50.522′ N, 49° 32.271′ E	620
**Rudbar**	Foothill	Rudbar	36° 49.314′ N, 49° 25.322′ E	270
**Klayeh**	Foothill	Rudbar	36° 50.992′ N, 49° 32.132′ E	438
**Rudabad**	Plain	Rudbar	36° 52.397′ N, 49° 30.871′ E	192
**Harkian**	Foothill	Rudbar	36° 59.592′ N, 49° 33.491′ E	149
**Siahroodposhteh**	Foothill	Rudbar	36° 59.862′ N, 49° 33.432′ E	269
**Upper Harzavil (Manjil)**	Foothill	Rudbar	36° 44.495′ N, 49° 26.072′ E	506
**Lower Harzavil (Manjil)**	Foothill	Rudbar	36° 44.837′ N, 49° 25.735′ E	453
**Halaj (Loshan)**	Foothill	Rudbar	36° 40.306′ N, 49° 26.792′ E	307
**Kacha**	Foothill	Rasht	37° 05.173′ N, 49° 36.973′ E	124
**Saghalaksar**	Plain	Rasht	37° 09.596′ N, 49° 31.334′ E	53
**Ghazian**	Coastal	Anzali	37° 27.347′ N, 49° 28.663′ E	−21
**Kandbon**	Plain	Rudsar	37° 03.415′ N, 50° 20.987′ E	20
**Kalesara**	Foothill	Talish	37° 42.251′ N, 48° 55.577′ E	93
**Eivazmahaleh**	Foothill	Astara	38° 23.964′ N, 48° 46.715′ E	77
**Sechekeh**	Foothill	Siahkal	37° 06.755′ N, 49° 50.985′ E	205
**Asooyebala (Tootaki)**	Mountainous	Siahkal	37° 03.556′ N, 49° 52.542′ E	355

**Table 2.** The meteorological data of the fixed site (Pareh Village of Rudbar County), Guilan Province, Iran, 2016

**Meteorological data**	**April**	**May**	**June**	**July**	**Aug**	**September**	**October**	**November**
**Maximum Temperature**	20.88	26.71	30.00	29.68	33.53	27.94	20.64	14.91
**Minimum Temperature**	10.79	16.56	20.17	21.52	22.60	18.77	12.92	6.25
**Average Temperature**	15.84	21.64	25.08	25.60	28.06	23.36	16.78	10.58
**Relative Humidity**	69.06	67.04	59.58	65.86	56.53	67.63	72.65	67.39
**Rainfall**	40.34	17.89	10.73	36.95	2.22	56.71	58.87	73.30

### Determining species dominance structure

The dominance structure of a species is expressed as the percentage of specimens of the species in the whole sample. The following five percentage representation categories (52, 53) were used: Eudominat (ED) species (> 30%), dominant (D) (10–30%), subdominant (SD) (5–10%), recedent (R) (1–5%) and subrecedent (SR) (< 1%).

### Mapping collected mosquitoes and statistical analysis

ArcGIS 10.3 was used to create a geo-database of mosquitoes and to map the collection sites and the distributions of the most medically important species. The statistical analysis of mosquito abundance and meteorological data was carried out using the linear regression test of SPSS software (ver. 16 for Windows, SPSS Inc., Chicago, IL).

## Results

### Mosquito fauna

Overall, 16327 adult mosquitoes were collected during 29 surveys (Aug–Dec 2015 and Apr–Oct 2016): 15959 (97.75%) were captured using light traps and 368 (2.25%) by ad hoc hand catches (Table 3). Eighteen species representing seven genera were identified morphologically: *Anopheles* (*Anopheles*) *claviger* (Meigen), *An.* (*Ano.*) *hyrcanus* (Pallas), *An.* (*Ano.*) *maculipennis* Meigen s.l., *An.* (*Ano.*) *pseudopictus* Grassi, *An.* (*Cellia*) *superpictus*, *Aedes* (*Aedimorphus*) *vexans* (*Meigen*) [*Aedimorphus vexans*], *Ae.* (*Dahliana*) *geniculatus* (Olivier) [*Dahliana geniculata*], *Ae.* (*Ochlerotatus*) *caspius* s.l. [*Ochlerotatus caspius* s.l.], *Ae.* (*Och.*) *pulcritarsis* (Rondani) [*Oc. pulcritarsis*], *Coquillettidia* (*Coquillettidia*) *richiardii* (Ficalbi), *Cx.* (*Culex*) *mimeticus* Noè, *Cx.* (*Cux.*) *perexiguus* Theobald, *Cx.* (*Cux.*) *pipiens*, *Cx.* (*Cux.*) *theileri*, *Cx.* (*Cux.*) *tritaeniorhynchus* Giles, *Culiseta* (*Culiseta*) *annulata* (Schrank), *Orthopodomyia pulcripalpis* and *Uranotaenia* (*Pseudoficalbia*) *unguiculata* Edwards (Table 3). *Aedes pulcritarsis* and *Cx. perexiguus* were found for the first time in Guilan Province.

**Table 3.** The collection method and composition of adult mosquitoes collected in Guilan Province, Iran, August–December 2015 and April–October 2016

**Species**	**Light trap**		**Hand catch**		**Total**		**Dominance structure**

	**n**	**%**	**n**	**%**	**n**	**%**	
***An. claviger***	18	0.11	2	0.54	20	0.12	Subrecedent
***An. hyrcanus***	29	0.18	-	-	29	0.17	Subrecedent
***An. maculipennis* s.l.**	752	4.71	144	39.13	896	5.48	Subdominant
***An. pseudopictus***	1760	11.03	23	6.25	1783	10.92	Dominant
***An. superpictus***	6	0.04	-	-	6	0.04	Subrecedent
***Ae. caspius* s.l.**	96	0.60	-	-	96	0.60	Subrecedent
***Ae. geniculatus***	1	0.01	-	-	1	0.01	Subrecedent
***Ae. pulcritarsis***	7	0.04	-	-	7	0.04	Subrecedent
***Ae. vexans***	2930	18.36	39	10.59	2969	18.18	Dominant
***Cq. richiardii***	94	0.59	1	0.28	95	0.60	Subrecedent
***Cx. mimeticus***	8	0.05	1	0.28	9	0.06	Subrecedent
***Cx. perexiguus***	6	0.04	-	-	6	0.04	Subrecedent
***Cx. pipiens***	3030	18.98	134	36.41	3164	19.37	Dominant
***Cx. theileri***	3844	24.09	9	2.44	3853	23.59	Dominant
***Cx. tritaeniorhynchus***	3375	21.15	14	3.80	3389	20.75	Dominant
***Cs. annulata***	1	0.01	-	-	1	0.01	Subrecedent
***Or. pulcripalpis***	-	-	1	0.28	1	0.01	Subrecedent
***Ur. unguiculata***	2	0.01	-	-	2	0.01	Subrecedent

**Total**	**15959**	**100**	**368**	**100**	**16327**	**100**	

### Species dominance structure

**Fig. 2.**
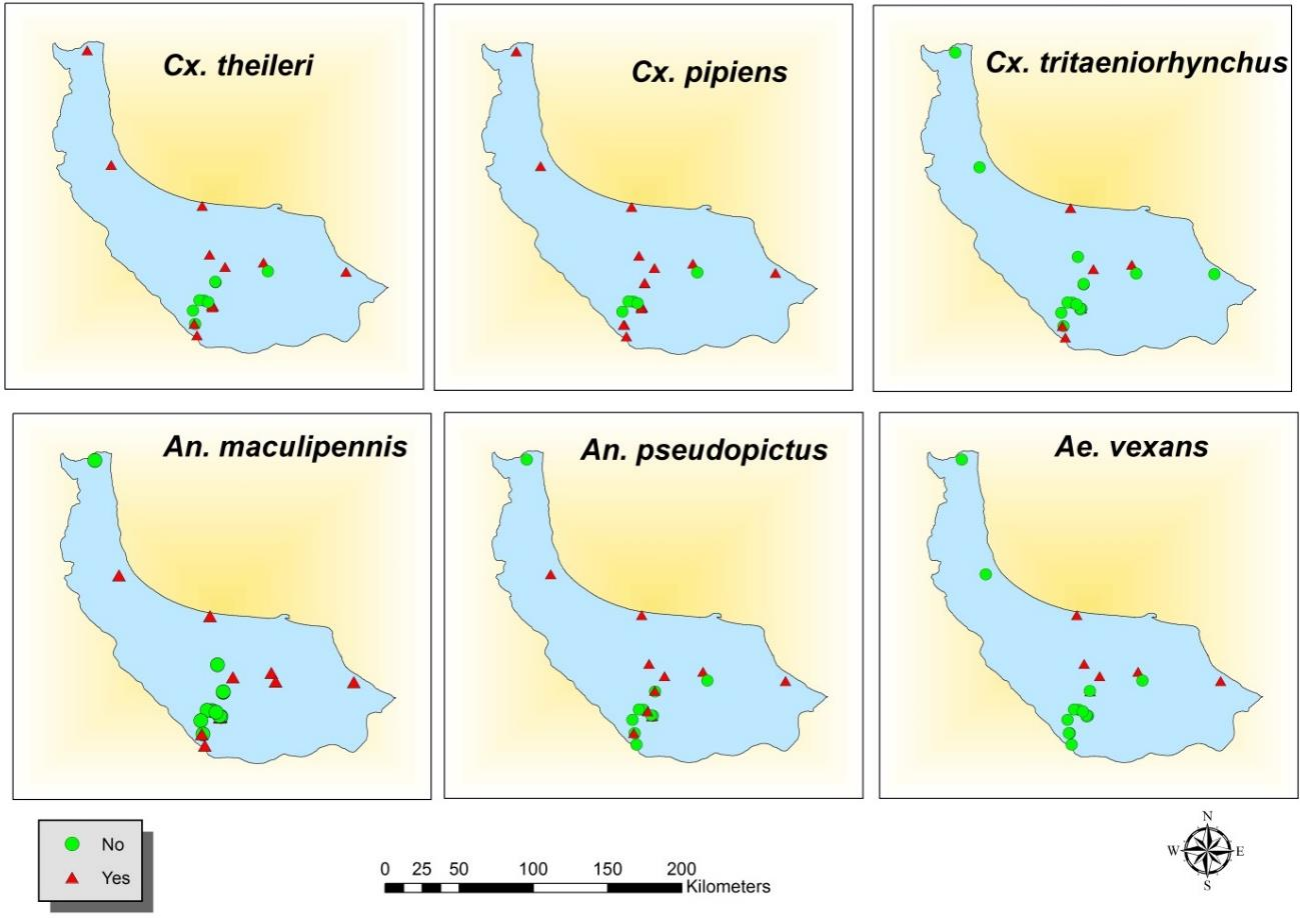
Distribution map of the most prevalent and medically important mosquitoes in the study areas in Guilan Province, Iran, 2015–2016

Overall, 2734 anopheline adults (16.71%) and 13,593 culicine adults (83.29%) were collected. The most prevalent species were *Cx. theileri* (23.59%, dominant), *Cx. tritaeniorhynchus* (20.75%, dominant), *Cx. pipiens* (19.37%, dominant), *Ae. vexans* (18.18%, dominant), *An. pseudopictus* (10.92%, dominant) and *An. maculipennis* s.l. (5.48%, subdominant). These six species included 16,054 specimens (98.1%) of the whole sample (Table 3). Moreover, they showed the widest distributions in the province (Table 4, Fig. 2). Regarding the dominance structure of subfamily Anophelinae, *An. pseudopictus* and *An. maculipennis* s.l. with the abundance percentages of 65.2% and 32.8% respectively, were both eudominant. In the case of percentage representation of subfamily Culicinae, *Cx. theileri* (28.34%), *Cx. tritaeniorhynchus* (24.93%), *Cx. pipiens* (23.27%) and *Ae. vexans* (21.84%) were dominant, as in the case of all mosquitoes, *i.e.* the total for both subfamilies.

**Table 4.** The distribution of adult mosquitoes collected in different counties in Guilan Province, Iran, August–December 2015 and April–October 2016

**Locality**	**County**						

**Species**	**Rudbar**	**Rasht**	**Anzali**	**Rudsar**	**Talish**	**Astara**	**Siahkal**
***An. claviger***	*	-	-	-	-	-	-
***An. hyrcanus***	-	*	-	*	-	-	-
***An. maculipennis* s.l.**	*	*	*	*	*	-	*
***An. pseudopictus***	*	*	*	*	*	-	*
***An. superpictus***	*	-	-	-	-	-	-
***Ae. caspius* s.l.**	*	-	*	-	-	-	-
***Ae. geniculatus***	*	-	-	-	-	-	-
***Ae. pulcritarsis***	*	-	-	-	-	-	*
***Ae. vexans***	*	*	*	*	-	-	*
***Cq. richiardii***	-	-	*	-	-	-	-
***Cx. mimeticus***	*	-	-	*	-	-	-
***Cx. perexiguus***	*	-	-	-	-	-	-
***Cx. pipiens***	*	*	*	*	*	*	*
***Cx. theileri***	*	*	*	-	-	-	*
***Cx. tritaeniorhynchus***	*	*	*	*	*	*	*
***Cs. annulata***	-	*	-	-	-	-	-
***Or. pulcripalpis***	*	-	-	-	-	-	-
***Ur. unguiculata***	*	-	-	-	-	-	-

### Seasonal activity and the fluctuations of rainfall and temperature

**Fig. 3.**
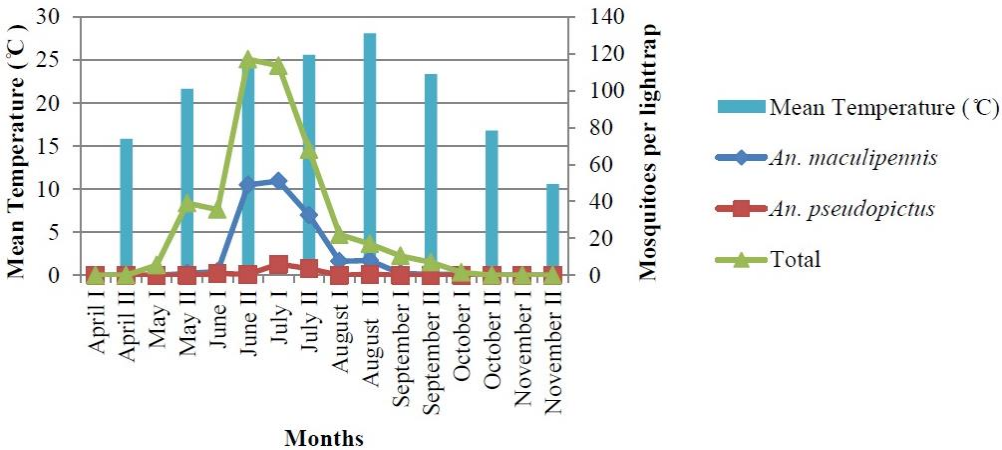
Biweekly abundance of the most prevalent anopheline mosquitoes and monthly mean temperature in the fixed site, Pareh Village of Rudbar County, Guilan Province, Iran, April–October 2016 (Total includes *Anopheles claviger*, *An. maculipennis* s.l., *An. pseudopictus*, *An. superpictus*, *Ae. pulcritarsis*, *Culex mimeticus*, *Cx. pipiens*, *Cx. theileri*, *Cx. tritaeniorhynchus*)

**Fig. 4.**
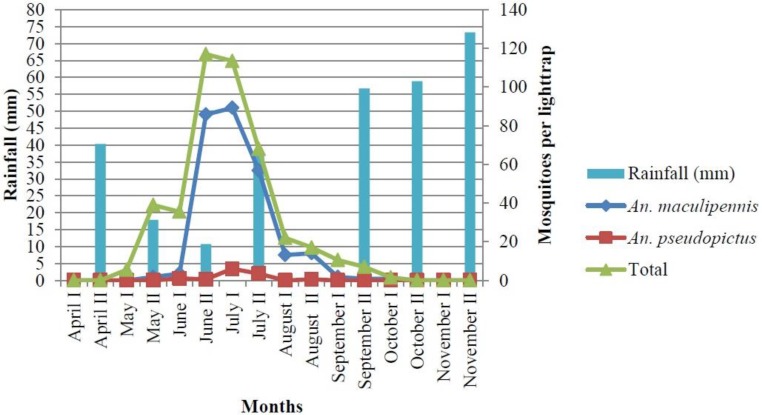
Biweekly abundance of the most prevalent anopheline mosquitoes and monthly rainfall in the fixed site, Pareh Village of Rudbar County, Guilan Province, Iran, April–October 2016 (Total includes *Anopheles claviger*, *An. maculipennis* s.l., *An. pseudopictus*, *An. superpictus*, *Ae. pulcritarsis*, *Culex mimeticus*, *Cx. pipiens*, *Cx. theileri*, *Cx. tritaeniorhynchus*)

**Fig. 5.**
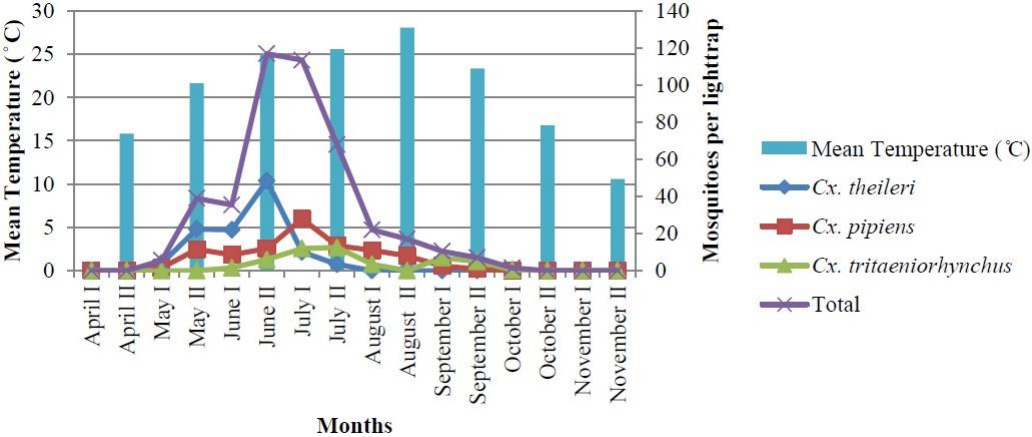
Biweekly abundance of the most prevalent culicine mosquitoes and monthly mean temperature in the fixed site, Pareh Village of Rudbar County, Guilan Province, Iran, April–October 2016 (Total includes *Anopheles claviger*, *An. maculipennis* s.l., *An. pseudopictus*, *An. superpictus*, *Ae. pulcritarsis*, *Culex mimeticus*, *Cx. pipiens*, *Cx. theileri*, *Cx. tritaeniorhynchus*)

**Fig. 6.**
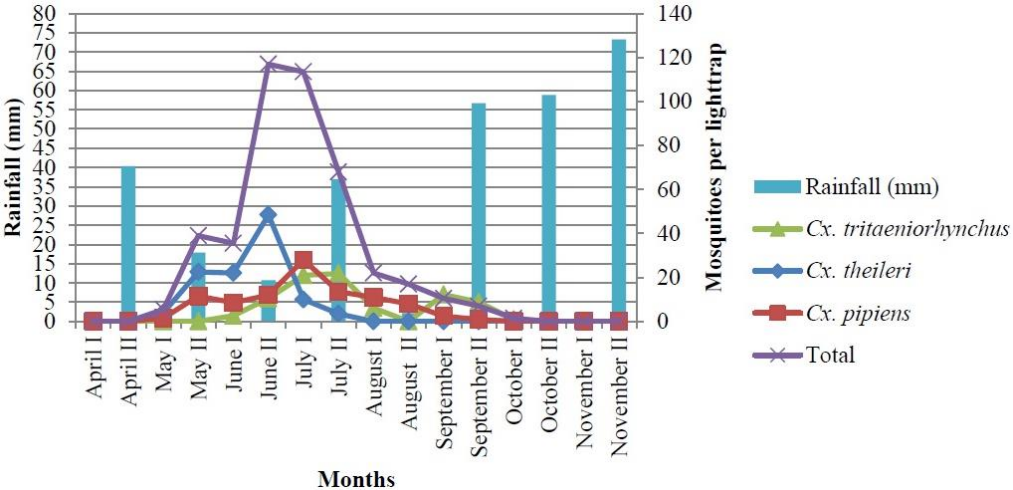
Biweekly abundance of the most prevalent culicine mosquitoes and monthly rainfall in the fixed site, Pareh Village of Rudbar County, Guilan Province, Iran, April–October 2016 (Total includes *Anopheles claviger*, *An. maculipennis* s.l., *An. pseudopictus*, *An. superpictus*, *Ae. pulcritarsis*, *Culex mimeticus*, *Cx. pipiens*, *Cx. theileri*, *Cx. tritaeniorhynchus*)

In general, the active season of adult mosquitoes extended from early May to early Oct in the fixed site (Pareh Village of Rudbar County). The peak of activity was late June for *Cx. theileri*, mid-July for *An. maculipennis* s.l., *An. pseudopictus* and *Cx. pipiens*, and late July for *Cx. tritaeniorhynchus*. While the peak of activity of most adult mosquitoes was late June to mid-July, and the abundance dramatically decreased after that, the monthly mean temperature increased by Aug. Also after Apr rainfall decreased in the fixed site during Jun and Jul and the rainy season started in Sep (Figs. 3–6). There is no significant regression between the abundance of adult mosquitoes and the meteorological data (Table 2) during active season in the fixed site (P> 0.05, R^2^= 0.31).

## Discussion

### Mosquito fauna

During the present investigation, 18 species representing seven genera of mosquitoes were collected in Guilan Province in which *Ae. pulcritarsis* and *Cx. perexiguus* were new records for the province. Thus, the number of species recorded in the province increased from 30 (36) to 33 (35 and the present investigation).

Seven species, *An.* (*Ano.*) *plumbeus* Stephens, *Ae.* (*Dah.*) *echinus* (Edwards) [*Dahliana echinus*], *Cx.* (*Maillotia*) *hortensis* Ficalbi, *Cx.* (*Cux.*) *torrentium* Martini, *Cx.* (*Neoculex*) *territans* Walker, *Cs.* (*Allotheobaldia*) *longiareolata* (Macquart) and *Cs. (Culicella) morsitans* (Theobald) whose larvae were collected during recent years in Guilan Province (36), were not captured in the present study. The reason is the rarity of some aforementioned species and the tendency of some, such as *Cx. hortensis*, *Cx. territans* and *Cs. morsitans*, to feed on birds, amphibians or reptiles (54), thus they were not attracted to light traps used in animal (cattle and sheep) shelters during the study.

Also, there are seven species of the Maculipennis Group in Guilan Province differentiated by egg patterns or the polymerase chain reaction (PCR) technique (36), which could not be differentiated with the morphology-based keys of females and larvae (29) used in the present study.

Among the species collected, *Ae. caspius* s.l. is known to vector WNV in West Azerbaijan Province in northwestern Iran (14). Shahhosseini et al. (55) referred to the virus later as Kunjin-related West Nile Virus. Moreover, WNV was found in *Cx. pipiens* in the Sepid-Rud valley of Guilan Province (15). In addition, *An. maculipennis* s.l., *Ae. vexans*, *Cq. richiardii*, *Cx. perexiguus*, *Cx. theileri*, *Cx. tritaeniorhynchus* and *Ur. unguiculata* is believed to play role as vectors of WNV in different countries of the Old World (1, 56). Among the aforementioned species, *Cq. richiardii* and *Cx. pipiens* in Europe and *Cx. tritaeniorhynchus* in Asia are the main vectors of the virus (1).

*Culex theileri* has been found to be the vector of *Dirofilaria*, the causal agent of dirofilariasis, in Ardebil Province in northwestern Iran (27), and *An. claviger*, *An. hyrcanus*, *An. maculipennis*, *An. pseudopictus*, *An. superpictus*, *Ae. vexans*, *Ae. caspius*, *Ae. geniculatus*, *Cq. richiardii*, *Cx. pipiens*, *Cx. tritaeniorhynchus*, *Cs. annulata* and *Ur. unguiculata* are known the vectors of *Dirofilaria* in different countries of the western Palaearctic Region (54, 57–59).

### Species dominance structure

In the present study, the most abundant species were *Cx. theileri*, *Cx. tritaeniorhynchus*, *Cx. pipiens*, *Ae. vexans*, *An. pseudopictus* and *An. maculipennis* s.l., respectively. With the exception of *An. maculipennis* s.l., which is subdominant, they are all dominant according to the classification (52, 53) (Table 3). This is concordant with the previous findings in the province based on collections of larvae (36–38), as well as adults (37, 60). The exception is *Cx. theileri*. The species had been found less often in the larval stage than any other *Culex* in the province (38). One reason is probably due to sampling, the heavy rainfall in the province results in a great number of different natural larval habitats that are favorable for *Cx. theileri* (38), but those habitats are not easily located and sampled. On the other hand, the favorable larval habitats of some species, such as *Cx. pipiens* and *Cx. tritaeniorhynchus*, i.e. artificial containers and rice fields, respectively, are easier to find and sample. Another reason is probably the biology of the species, some species such as *Cx. hortensis* and *Cx. territans*, which do not bite humans and mammals but mostly feed on amphibians, reptiles or birds (54), have been collected very often as larvae (38). They were not collected during the present investigation by means of aspirators and light traps from animal shelters which attract *Cx. theileri* (Table 3). The most prevalent species of the province, *An. maculipennis* s.l., *An. pseudopictus*, *Ae. vexans*, *Cx. pipiens*, *Cx. theileri* and *Cx. tritaeniorhynchus* are known vectors of both WNV and *Dirofilaria* (1, 57, 59).

### Seasonal activity and the fluctuations of rainfall and temperature

During the present investigation, *Ae. vexans* was one of most abundant species and most prevalent aedine species (Table 3), as noted previously (37, 60). However, most specimens were collected from Anzali (Table 4) and the species was not collected from the fixed site, so its seasonal activity is not discussed here. *Anopheles maculipennis* s.l. showed the peak of activity in the mid-Jul (Figs. 3, 4). The peak of monthly activity of anophelines (including *An. maculipennis* s.l. and *An. superpictus*) was reported during Jul–Aug in Kalaleh County of Golestan Province, northern Iran (47). The most *An. maculipennis* s.l. was captured in Aug in Aras Valley, Turkey, adjoining Iran (61). There are no records for the seasonal activity of culicine adults in Iran. The peak of activity of *Cx. pipiens* was found in Jul in northern Italy (62). That is in concordance with the result of the present study (Figs. 5, 6). However, the peak of activity of *Cx. pipiens* was recorded in Aug in the Belek Region and Aras Valley of Turkey (61, 63). Most *Cx. theileri* was found in Jun in Aras Valley of Turkey (61), which is similar to the present study (Figs. 5, 6), however, the peak activity of this species was reported in Aug in Ankara, Turkey (64). Moreover, most *Cx. tritaeniorhynchus* was captured in Aug in Belek Region of Turkey (63), while the peak of activity was observed in Jul in the present study (Figs. 5, 6). Differences between the results of the present investigation and the findings in other regions may be explained by differences in the topography and climates (especially temperature) which influence the bionomics of mosquitoes. On the other hand, some differences are due to sampling regimes. For example, mosquito abundance was reported based on weekly catches (62), and on monthly catches (61, 63, 64), whereas the seasonal activity is based on biweekly captures in the present study and another study (47). Besides, dry-ice baited traps was used for sampling by Alten et al. (63), BG-traps by Roiz et al. (62) and spray sheet collections by Sofizadeh et al. (47), whereas light traps were used by Simsek (64), Alkan and Aldemir (61) and in this study.

Though the mean monthly temperature of about 16 °C is a limiting factor in the activity of adult mosquitoes in the study area (Figs. 3, 5), no significant regression was observed between different meteorological data (Table 2) and the abundance of adult mosquitoes during active season (P> 0.05, R^2^= 0.31). During the present study, the peak of activity of most adult mosquitoes was late Jun to mid-Jul, only the peak of activity of *Cx. tritaeniorhynchus* was in late Jul (Figs. 3–6), after which the abundance dramatically decreased as temperature increased by Aug (Figs. 3, 5). High temperature (> 35 °C) is generally a limiting factor for the abundance of adult mosquitoes, especially in localities with warm climate such as southern Iran (41) and Saudi Arabia (65). However, it does not seem that temperature was a key factor in decreasing the abundance of adult mosquitoes in the area of the present study, because the temperature does not exceed 34 °C and the monthly mean temperature is lower than 30 °C in the fixed site in Aug (Figs. 3, 5). In addition, rainfall decreased in the fixed site during Jun and Jul while the abundance increased. The rainy season started in Sep while the abundance of mosquitoes dramatically decreased (Figs. 4, 6). A key factor mentioned here is rice fields, the main larval habitats of the most prevalent species, are dry during Aug. Temperature decreases significantly during Sep and Oct, consequently, the prevalence of mosquitoes decreases.

In view of integrated vector management, ecological data, especially seasonal activity, is very important for intervention measurements. On the other hand, one of main intervention measurements is using pesticides yet. There is little-published data about the susceptibility status of mosquitoes, especially culicines (66, 67), in northern Iran. This subject can be a goal for forthcoming studies in Guilan Province.

## Conclusion

Though there was no significant regression between the abundance of adult mosquitoes and the meteorological data in the fixed site during active season, temperature and rice fields had a great influence in starting and ending active season in the region. The seasonal activity of the important species *Ae. vexans*, other species found less abundant in this study, host preference analysis and filarial and arbovirus screening should be the subjects of future investigations in the region.
